# The Activity of Antimicrobial Peptides in Pediatric Celiac Disease

**DOI:** 10.3389/fped.2022.873793

**Published:** 2022-06-06

**Authors:** Altinoy T. Kamilova, Gulnoza K. Azizova, Zulkhumar E. Umarnazarova, Dilrabo A. Abdullaeva, Svetlana I. Geller

**Affiliations:** Gastroenterology Department of Republican Specialized Scientific-Practical Medical Center of Pediatrics Ministry of Health of Republic of Uzbekistan, Tashkent, Uzbekistan

**Keywords:** celiac disease, children, antimicrobial peptides, β-defensin 2, fecal calprotectin, bactericidal permeability increasing protein

## Abstract

**Background:**

Celiac disease (CD) is an immune-mediated disorder of the gut in which innate and adaptive responses are involved. Antimicrobial peptides (AMPs) constitute an arsenal of innate immunity regulators of paramount importance in the gut. However, the role of AMPs in CD is unclear.

**Aims:**

To evaluate the levels of fecal β-defensin-2, fecal calprotectin (FC), and antibodies against bactericidal/permeability-increasing protein (BPI) in the serum of children with active CD and to compare them with those of healthy controls (HCs).

**Methods:**

We examined 76 children with recently diagnosed CD between the age of 2–10 years (average age: 6.1 ± 1.2 years) and 32 HC (average age: 6.2 ± 3.8 years) in this study. We evaluated the level of fecal β-defensin-2 and FC levels in coprofiltrates, and the level of anti-BPI antibodies in blood serum. Correlation relationships between the parameters were assessed according to Pearson correlation coefficient.

**Results:**

Fecal β-defensin-2 concentration was greater in the CD group than in HC group, amounting to 99.6 ± 15.5 ng/mL and 64.0 ± 2.4 ng/mL, respectively (*p* < 0.02). The level of FC in the CD children was 35.4 ± 8.1 μg/g, while that in the control group was 19.1 ± 1.1 μg/g, (*p* < 0.05), representing a slightly increase. The concentration of anti-BPI antibodies in the CD and HC groups was 35.9 ± 10.1 U/mL and 5.2 ± 3.2 U/mL, respectively (*p* < 0.002). There was a strong and direct correlation between fecal β-defensin-2 and FC (*r* = 0.69), as well as a direct but weak relationship between fecal β-defensin-2 and anti-BPI antibodies (*r* = 0.35).

**Conclusions:**

Our data reinforce that fecal β-defensin-2 and anti-BPI antibodies are greatly increased in patients with active CD. These biomarkers may be components of epithelial innate immunity in the intestine, with each having a distinct functional role in intestinal6 mucosal defense.

Celiac disease (CD) is an immune-mediated systemic disease characterized by atrophic enteropathy, which can manifest as a spectrum of both gastrointestinal and extraintestinal symptoms in genetically predisposed individuals (1).

CD occurs in approximately 1% of the population; previously, it was reported to be more prevalent in European individuals, however, recent studies have demonstrated a similar prevalence in Asian individuals (2, 3).

CD is a T cell disease in which peptides derived from gliadin, in native form or deaminized by transglutaminase, activate the lamina, leading to inflammatory responses of the adaptive immune system (4).

The role of the innate immune response in the development of CD has been attributed to gliadin. Researchers believe that gliadin fragmentation influences *in situ* recognition of the dominant gliadin epitopes by T cells and creates a proinflammatory environment necessary for subsequent T cell activation and tissue destruction (5).

The composition of the intestinal microbiota of patients with CD is altered compared to that of healthy controls (HCs) and is thought to contribute to the pathogenesis of CD (6–10). In particular, studies have shown a decrease in the number of beneficial bacteria, such as *Faecalibacterium prausnitzii* and *Bifidobacterium longum*, in untreated CD patients (11).

At the same time, there is evidence of disruption of the gut microbiome before the manifestation of CD.

Study by Leonard et al. showed significant changes in the gut microbiota and associated metabolomes prior to the onset of CD. Some strains and their metabolites, which have previously been associated with inflammatory and autoimmune processes, have been found to increase in numbers prior to disease manifestation (12). The authors also identified a decline in some pre-celiac microbiota variants that were previously reported to have anti-inflammatory properties. In addition, several other metabolites have been identified that have not been previously reported and are possibly associated with CD. These are the increased number of bacteria *D. invisus, Parabacteroides sp.*, and *L. bacterium* in CD. The revealed changes indicate the transition from the preclinical stage of the disease to impaired gluten tolerance and the subsequent development of CD and can serve as microbial markers of disease progression toward the onset.

Therefore, an increase in the strains of microorganisms associated with inflammatory and autoimmune processes has been reported to cause impaired gluten tolerance in the preclinical stage of the disease.

The innate immune system includes such an important element as antimicrobial peptides (AMPs) present on various surfaces of the human body, as well as in human neutrophils, monocytes and lymphocytes (13).

AMPs have been identified as key regulators of interactions between commensal microbes and host tissues (14). The best-known function is antimicrobial activity against invading microorganisms including bacteria, fungi and enveloped viruses (15–17) and exhibit other biological functions such as lipopolysaccharide (LPS) neutralization, wound healing, chemotactic activity, and immunomodulation of epithelial surfaces (18–20).

In addition to these direct antimicrobial activities, AMPs have also been found to play essential roles in shaping the composition of the local microbiome (21).

It has now been established that specific producers of β-defensins 2 in the colon are mucosal enterocytes, macrophages, and dendritic cells (22). Given the fact that β-defensins 2 are synthesized by epitheliocytes of the mucosa of the gastrointestinal tract, including the stomach, in response to any damaging factor, it is assumed that it can be used as a regional molecular marker of inflammation of the mucous membrane of the upper intestine (18). In particular, there is an increase in fecal beta-defensin 2 in inflammatory bowel disease (IBD) (23).

Fecal calprotectin (FC) is a calcium-binding heterodimer of the S100 protein family present in human and other mammalian granulocytes, macrophages, and epithelial cells (24). FC is released upon neutrophil/monocyte activation and can be found in serum and body fluids, including stool (25). The effectiveness of FC as a laboratory marker is being investigated in IBD in assessing endoscopic disease activity and predicting disease recurrence and response to treatment (26).

Bactericidal/permeability-increasing protein (BPI) is a neutrophil-derived cationic protein with bactericidal activity toward Gram-negative bacteria (27, 28) and is known for its potent anti-inflammatory, LPS-neutralizing activity (29).

The AMPs secreted by the cells of the intestinal wall activate innate immune mechanisms, but their involvement in malabsorption-based intestinal diseases is not clear, further outlining the importance of this research.

The aim of this study was to evaluate the levels of fecal β-defensin-2, FC, and anti- BPI antibodies in the serum of children with active CD and compare them to those in HC.

## Methods

### Study Design

The study was performed prospectively from January 2018 to June 2019 at the Gastroenterology Department of Republican Specialized Scientific-Practical Medical Center of Pediatrics (RSSPMCP), Tashkent, Uzbekistan.

Inclusion Criteria: children aged 2–10 years with celiac disease and healthy children of the same age; the possibility of prospective monitoring of patients (monitoring the condition and adherence to the diet of children with a previously established diagnosis).

Exclusion Criteria: -the presence of infectious and parasitic diseases (giardiasis, amoebiasis); presence of IBD (ulcerative colitis, Crohn's disease); having irritable bowel syndrome.

Controls were recruited from the community and included healthy volunteers with no known history of gastrointestinal diseases or symptoms per Rome IV, 32 children aged 2–10 years.

### Ethical Statement

The study was conducted according to the standards of bioethics and was approved by the ethical committee of the RSSPMCP (approval no. IP-2018-1223). Informed written consent was acquired from their parents or guardians and the research was conducted in compliance with the World Medical Association Declaration of Helsinki.

### Clinical Data

For data collection, the case history of each patient with CD was used, which included name, date of birth, current height and weight, and information about intermittent abdominal pain, constipation, diarrhea, known chronic diseases, and family history. All patients underwent physical examination, general and biochemical blood tests, general urinalysis and stool examination for hidden blood and parasites, abdominal ultrasound, and determination of total protein, calcium, alanine transaminase (ALT), aspartate aminotransferase (AST), bilirubin, and alkaline phosphatase levels (ALP). If clinically indicated, other paraclinical examinations were performed, such as functional tests of the thyroid gland, colonoscopy, multi- slice computed tomography, and barium enema.

### Laboratory Analysis

CD diagnosis was confirmed on the basis of an increased titer of anti-tissue transglutaminase (tTg) immunoglobulin (Ig) A antibody compared to the normal values of total IgA Orgentec Diagnostika GmbH Enzyme-Linked Immunosorbent Assay (ELISA) kit for quantitative determination in human serum, Cat. No. 416-5400A. According to the 2012 European Society for Pediatric Gastroenterology, Hepatology and Nutrition (ESPGHAN) guidelines, a no-biopsy pathway for symptomatic children with anti-tTg IgA values ≥10 times the upper limit of normal with appropriate tests and positive endomysial antibodies (EMA) IgA in a second serum sample (30–32). Since we could not analyze EMA IgA all serologically positive patients (*n* = 76) underwent to upper gastrointestinal endoscopy and duodenal biopsy (by Pentax EG2930K endoscope after overnight fasting). The biopsy samples were included in neutral buffered formalin and processed according to standard procedures, in order to be evaluated by two experienced pathologists who graded the histologic findings according to the modified Marsh criteria (33).

Physical development of the children was assessed according to the World Health Organization (WHO) guidelines (34).

Fecal β-defensin-2 (Immundiagnostics, Cat. No.K 6500) and FC levels (Buhlmann, Cat. No. EK-CAL) were measured with an ELISA kit (Immundiagnostics). A one-step sandwich version of solid-phase enzyme immunoassay was used. Stool samples after pre-treatment with special extraction buffer were frozen and stored at −20°C. The level of anti-BPI antibodies was determined in the serum using a chemiluminescent immunoassay (CLIA) kit (Orgentec Diagnostika GmbH, Cat. No. 523), which involves an indirect enzyme-linked immune reaction. Serum samples were stored frozen at −20°C.

### Statistical Analysis

Statistical analyses of the obtained data was performed using Microsoft Excel with a library of statistical functions and STATISTICA10.0 [StatSoft, Inc. (2011)], including methods of mathematical statistics, in particular, relative values (frequency, %). We used the methods of variation statistics—arithmetic mean (M), standard deviation (σ), standard error (*m*). analysis of variance—Student's test (*t*), and probability of error (*p*). Differences in the mean values were considered significant if *p* ≤ 0.05. Correlation relationships between the parameters were assessed according to Pearson correlation coefficient. Descriptive statistics are presented as numbers and percentage (%) for qualitative variables and as average ± standard deviation for quantitative variables.

## Results

In this study, we included 76 children, 54 girls (71.0%) and 22 boys (29.0%) with recently diagnosed CD (no more than one year from the date of the study) between the age of 2 to 10 years (average age: 6.1 ± 1.2 years) and 32 HCs (19 girls (59.3%) and 13 boys (40.4%), average age: 6.2 ± 3.8 years.

We observed that the most common symptom of CD was low weight (85.5%). The Z-score of different parameters were as follows: height relative to age, −1.94 (−3.94; −0.06); body weight relative to age, −1.36 (−4.44; 1.17); body weight relative to height, −0.93 (−4.10; 2.76); and body mass index relative to age, −0.98 (−3.85; 2.72).

**Figure 1:**
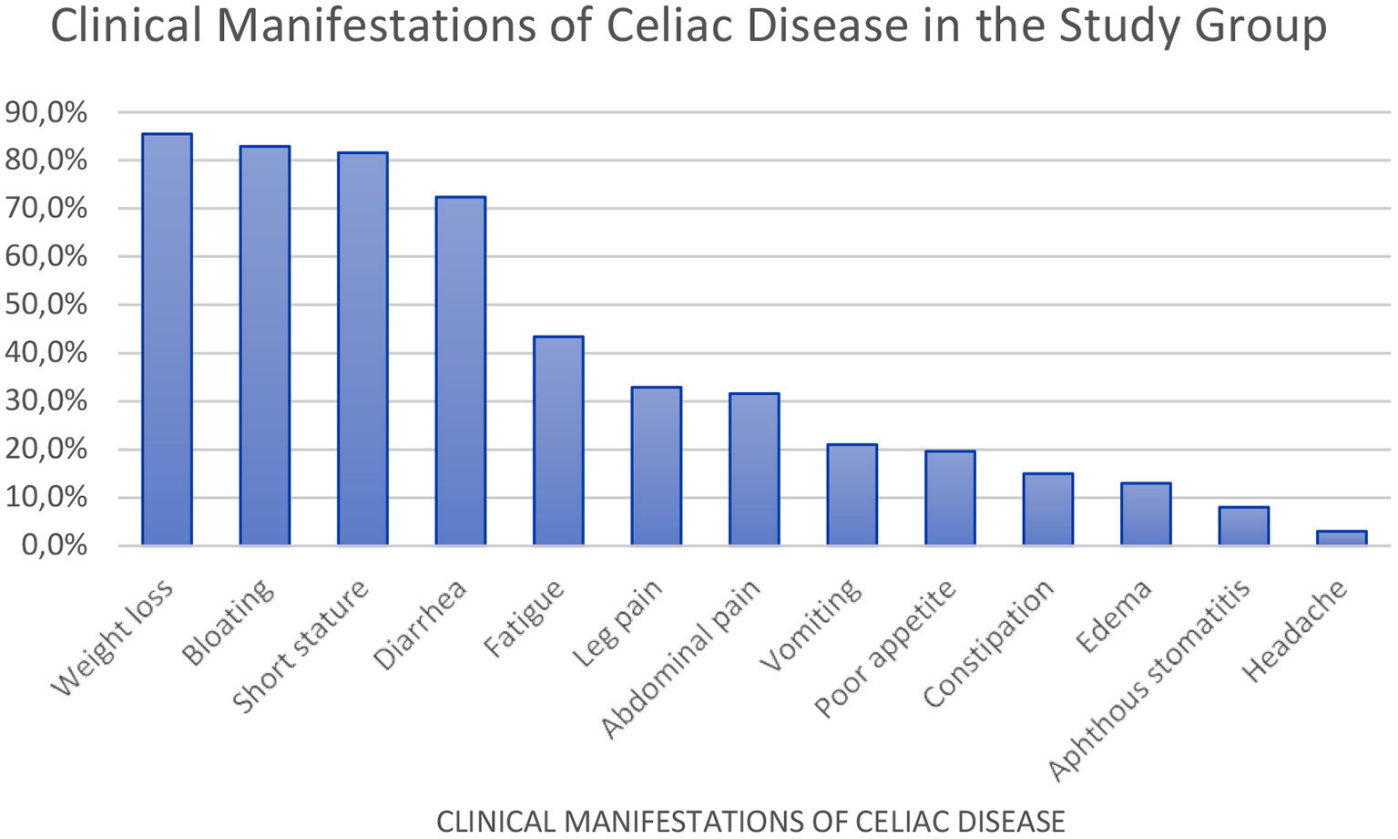
Clinical manifestations of celiac disease in patients, %.

Other observations in CD patients included abdominal bloating (82.9%), short stature (81.6%), diarrhea (72.4%), chronic fatigue (43.4%), leg pain (32.9%), and recurrent abdominal pain (31.6%) (Figure 1).

Classical (typical) symptoms of CD were observed in 54 (71%) patients, atypical manifestations in 22 (28.9%). The ratio of these forms of CD was 2.5:1.

The hemoglobin level in our CD patients was significantly decreased, and the level of alkaline phosphatase in the blood serum was increased (Table 1). The average of anti-tTG-IgA values was 10 times higher in CD patients, 171.7 ± 20.3 U/mL (Table 1). ALT values were also higher than those of HCs, but remained within the reference range (Table 1).

**Table 1:** Biochemical and serologic test-results of patients with celiac disease compared to those of healthy controls (*n* = 76).

**Signs**	**Sick children with CD *n =* 76**	**HCs *n =* 32**	***p*** **-value**
HGB, (g/L)	99.7, 3.1	115.6, 3.2	≤ 0.001
EOS, (%)	2.77, 0.17	2.0, 0.15	≥0.05
PLT, (10^9^/L)	294.16, 100.8	315, 85.9	≥0.05
WBC, (10^9^/L)	7.76, 3.16	7.0, 2.5	≥0.05
LYM, (%)	44.11, 10.26	40.2, 8.5	≥0.05
Ca, (mmol/L)	2.0, 0.12	2.3, 0.4	≥0.05
ALP, (U/L)	325.2, 69.9	139.25, 6.58	≤ 0.02
Anti tTG IgA, (U/mL)	171.7, 20.3	5.2, 2.6	≤ 0.001
ALT, (U/mL)	27.0, 2.1	17.8, 2.6	≤ 0.02
AST, (U/mL)	34.5, 2.7	20.1, 2.2	≥0.05

*P-validity between groups of patients with celiac disease and the control group. Counts (%), medians (interquartile range), and means ±SD are presented appropriate*.

*HGB, hemoglobin; EOS, eosinophils; PLT, platelets; WBC, white blood cells; LYM, Lymphocytes; Ca, calcium; ALP, alkaline phosphatase; Anti tTG IgA, anti-tissue transglutaminase IgA; ALT, alanine aminotransferase; AST, aspartate aminotransferase*.

Histological examination of mucosal biopsy specimens in majority of the patients revealed changes corresponding to Marsh III, the most frequent being complete villous atrophy (IIIc), indicating the duration and late diagnosis of the disease. In addition to complete villous atrophy, all biopsy specimens showed increased levels of intraepithelial lymphocytes (more than 40/100 epithelial cells) and infiltration with mononuclear cells (lymphocytes and plasma cells) in the mucosal plate (Table 2).

**Table 2:** Histological findings according to Marsh classification in patients with celiac disease (*n* = 76).

**Signs**	**Sick children with CD *n =* 76**
Marsh I infiltrative	7 (9.2%)
Marsh II hyperplastic	17 (22.4%)
Marsh III destructive:	52 (68.4%)
Marsh IIIa	2 (3.8%)
Marsh IIIb	19 (36.5%)
Marsh IIIc	31 (59.7%)

Fecal β-defensin-2 concentration was greater in the CD group than in HCs, amounting to 99.6 ± 15.5 ng/mL and 64.0 ± 2.4 ng/mL, respectively (*p* < 0.02; Table 3). FC level in the CD children was 35.4 ± 8.1 μg/g, while that in the HCs was 19.1 ± 1.1 μg/g, (*p* < 0.05), representing a slightly increase (Table 3). The level of anti-BPI antibodies in the CD and HC groups were 35.9 ± 10.1 U/mL and 5.2 ± 3.2 U/mL, respectively (*p* < 0.002; Table 3).

**Table 3:** Antimicrobial peptide values in children with celiac disease.

**Indicators**	**CD group *n =* 76**	**HC *n =* 20**	**References**	**p-value**
FC, μg/g	35.4 ± 8.1	19.95 ± 1.1	<50	≤ 0.05
Fecal β-defensin, ng/mL	99.6 ± 15.5	64.3 ± 2.4	8-60	≤ 0.02
BPI, U/mL	35.9 ± 10.1	5.2 ± 3.2	<10	≤ 0.002

There was a strong and direct correlation between fecal β-defensin-2 and FC (*r* = 0.69), as well as a direct but weak relationship between fecal β-defensin-2 and anti-BPI antibodies (*r* = 0.35).

## Discussion

The average age of patients with CD was 6.1 ± 1.2 years, which indicates a late diagnosis of CD, given the predominantly classical form of the disease in our patients. This determined a significant delay in physical development, the predominance of deep atrophic processes in the mucosa of the small intestine in the children we observed. Biochemical parameters revealed high values of ALP in patients with celiac disease, which we attribute to possible vitamin D deficiency, typical for patients with celiac disease in Uzbekistan. In our previous study, an inverse high correlation between vitamin D content and ALP level in celiac disease was established (35). High serum ALP levels were also noted by Indian scientists (36).

We found a high increase in antibodies to the bactericidal cell-permeability-increasing protein known for its potent anti-inflammatory LPS-neutralizing activity in serum (29, 37) and also, a statistically significant increase in fecal β-defensin in children with celiac disease. FC levels remained within the reference values. The findings suggest the need an additional treatment to gluten-free diet (GFD) in children with celiac disease. Defensins are the most abundant AMP associated with the intestinal mucosa (38).

The main producers of β-defensins in the intestine are mucosal enterocytes, macrophages, and dendritic cells. The participation of these cellular structures in inflammatory processes may lead to the rapid release of defensins, which is most likely a protective mechanism and is aimed at suppressing the activity of the intestinal bacterial flora (22). β-defensins are formed in the mucosal epithelium in all parts of the gastrointestinal tract, pancreatic and salivary glands, skin, and some leukocyte subpopulations (39). Since β-defensins 2 are synthesized by epitheliocytes of the gastrointestinal tract mucosa, including the stomach, in response to some damaging factor, they can be considered as molecular markers for inflammation of the upper intestinal mucosa (18). In the intestine, defensins control microbial attachment and penetration (40).

We could not find data on fecal β-defensin 2 levels in patients with CD in the available literature. However, Vordenbaumen et al. (41) determined that the expression level of β-defensin 2 in antral biopsy specimens from patients with active CD was markedly higher than that in HCs. Similar results were obtained by Boniotto et al. (42). High levels of β-defensin-2 in feces were also observed in our study. At the same time, Forsberg et al. and Taha et al. reported contradictory results; a decrease in β-defensin 2 level in duodenal biopsy specimens. (43–45).

These inconsistent results may be due to different study designs and patient numbers, so the mechanism and effects of β-defensin 2 in CD are yet to be determined; further research in this area is required.

BPI, a 55-60 kDa protein first reported in 1975, has multifunctional roles. For example, its distinguished role in neutralizing endotoxins is promising for patients with septic shock. However, research has shown that BPI not only neutralizes bacterial lipopolysaccharides, but has a variety of other functions (46).

The available literature lacks data on BPI and antibodies against BPI in patients with CD. However, our research has identified a significant increase in the levels of anti-BPI antibodies in children with newly diagnosed CD, suggesting there could be an increase in opportunistic flora in children with CD in the active phase of inflammation.

FC is secreted by activated neutrophils in inflammatory diseases of the gastrointestinal tract, such as ulcerative colitis and Crohn's disease. Carroccio et al. (47) reported increased concentrations of FC in patients with CD (>50 mg/g) in 5 of 10 adults and 6 of 13 children. Another study reported that children with CD had significantly higher FC values than those of HCs, which approached normal values after 4 weeks of a GFD (48). Canadian researchers have also shown that FC level was elevated in the active phase of CD, suggesting potentially higher mucosal inflammation in patients with CD (49).

Ertekin et al. (50) also stated that FC concentration is increased in children with CD and is associated with the severity of histopathological findings in biopsy specimens of upper mucosa and responds to a GFD. Berni et al. (48) proposed FC as a marker of dietary compliance in combination with other tests.

Balamtekin et al. (51) determined that FC concentrations were significantly higher in newly diagnosed patients with CD than in patients on a GFD or healthy children. Further, patients with typical gastrointestinal symptoms had higher FC levels than patients with extraintestinal symptoms.

In contrast to the results reported by the aforementioned studies, Montalto et al. (52, 53) found no statistically significant difference between adult patients with CD and controls, and found no correlation between FC concentration and histological severity of the lesion.

A study by Biskou et al. (54) in adult patients with CD showed no significant differences in median FC between treated and untreated CD. In children with refractory CD, median FC value was significantly higher than that of the HCs and in children with classical CD. At the same time, none of these children had high FC values (100 ng/g), which is in accordance with our study.

Moderately elevated FC in patients with CD is explained by a false elevation due to minor inflammation of the rectal mucosa as a result of impaired absorption of nutrients or passage of residual antigenic gliadin fragments through the colon and inflammation of the rectum (55, 56). In our study, the FC concentration in children with first-time diagnosis was higher than that of the HCs, but the difference was slight and the values were within the reference range.

Studies by Poddighe et al. show that barriers to diagnosis in Central Asia include low awareness of the disease among physicians and/or patients, limited access to diagnostic resources (due to economic and/or organizational and/or geographic reasons), misuse or misinterpretation of available serologic tests, lack of standardized diagnostic and endoscopic protocols, and insufficient experience with histopathologic interpretation (57).

Our research, conducted in a comparatively small number of children with CD, showed the diagnostic detection of this disease relatively late, which is associated with certain diagnostic barriers that exist in Uzbekistan (58). However, further studies will be necessary to fully study the activity of AMPs in children with CD, as well as to determine them in the dynamics for the effect of a GFD on their activity.

## Conclusion

Our study shows that levels of fecal β-defensin-2 and anti-BPI antibodies are highly increased in patients with active CD. These biomarkers may be components of epithelial innate immunity in the intestine, each occupying a distinct functional role in intestinal mucosal defense.

## Data Availability Statement

The original contributions presented in the study are included in the article/supplementary files, further inquiries can be directed to the corresponding author/s.

## Ethics Statement

The studies involving human participants were reviewed and approved by Republican Scientific Medical Center of Pediatrics. Written informed consent to participate in this study was provided by the participants' legal guardian/next of kin.

## Author Contributions

AK: study conception and design. GA and SG: data collection. AK, ZU, and DA: analysis and interpretation of results. GA and SG: draft manuscript preparation. All authors reviewed the results and approved the final version of the manuscript.

## Conflict of Interest

The authors declare that the research was conducted in the absence of any commercial or financial relationships that could be construed as a potential conflict of interest.

## Publisher's Note

All claims expressed in this article are solely those of the authors and do not necessarily represent those of their affiliated organizations, or those of the publisher, the editors and the reviewers. Any product that may be evaluated in this article, or claim that may be made by its manufacturer, is not guaranteed or endorsed by the publisher.
